# COVID-19 gastrointestinal manifestations: a systematic review

**DOI:** 10.1590/0037-8682-0714-2020

**Published:** 2020-11-25

**Authors:** Filipe Antônio França da Silva, Breno Bittencourt de Brito, Maria Luísa Cordeiro Santos, Hanna Santos Marques, Ronaldo Teixeira da Silva, Lorena Sousa de Carvalho, Elise Santos Vieira, Márcio Vasconcelos Oliveira, Fabrício Freire de Melo

**Affiliations:** 1 Universidade Federal da Bahia, Instituto Multidisciplinar em Saúde, Vitória da Conquista, BA, Brasil.; 2 Universidade Estadual do Sudoeste da Bahia, Vitória da Conquista, BA, Brasil.

**Keywords:** COVID-19, SARS-CoV-2, Gastrointestinal manifestation, Gastrointestinal symptom, Systematic review

## Abstract

**INTRODUCTION:**

The pandemic caused by severe acute respiratory syndrome coronavirus 2 (SARS-CoV-2) infection has greatly challenged public health worldwide. A growing number of studies have reported gastrointestinal (GI) symptoms. We performed a systematic review of GI symptoms associated with coronavirus disease 2019 (COVID-19) as well as of the serum levels of biomarkers related to liver function and lesion in SARS-CoV-2-infected individuals.

**METHODS:**

We surveyed relevant articles published in English, Spanish, and Portuguese up to July, 2020 in the PubMed, MEDLINE, SciELO, LILACS, and BVS databases. Moreover, we surveyed potentially important articles in journals such as the NEJM, JAMA, BMJ, Gut, and AJG.

**RESULTS:**

This systematic review included 43 studies, including 18,246 patients. Diarrhea was the most common GI symptom, affecting 11.5% of the patients, followed by nausea and vomiting (6.3%) and abdominal pain (2.3%). With regard to clinical severity, 17.5% of the patients were classified as severely ill, whereas 9.8% of them were considered to have a non-severe disease. Some studies showed increased aspartate transaminase and alanine aminotransferase levels in a portion of the 209 analyzed patients and two studies.

**CONCLUSIONS:**

Our results suggest that digestive symptoms are common in COVID-19 patients. In addition, alterations in cytolysis biomarkers could also be observed in a lesser proportion, calling attention to the possibility of hepatic involvement in SARS-CoV-2-infected individuals.

## INTRODUCTION

Respiratory syndrome coronavirus 2 (SARS-CoV-2) infection was first reported as a viral pneumonia outbreak in Wuhan, China, in December 2019, and its rapid spread has become a public health challenge^1^ ^,^ ^2^. The potentially fatal coronavirus disease 2019 (COVID-19) has evolved to a pandemic affecting all continents, except for Antarctica^2^ ^,^ ^3^. As at July 30, 2020, more than 16,812,763 cases and 662,095 deaths have been reported globally according to the World Health Organization (WHO)^4^. SARS-CoV-2 is an infectious agent associated with a large-spectrum clinical presentation^5^, which classically involves respiratory tract symptoms such as fever, dry cough, and shortness of breath. Myalgia and fatigue are also commonly reported, while taste and olfactory disorders are more common when associated with other manifestations^6^ ^,^ ^7^. Interestingly, a study published in January, 2020 reported a patient with diarrhea as a gastrointestinal (GI) manifestation of SARS-CoV-2 infection. Since then, several cases reporting COVID-19 along with GI symptoms such as diarrhea, nausea, vomiting, abdominal pain, anorexia, and GI bleeding have been described^8^. Among the GI symptoms that have been described in adult COVID-19 patients, the most common are diarrhea, followed by nausea/vomiting and abdominal pain, while in pediatric patients, vomiting is more frequently reported^3^ ^,^ ^9^. In addition, studies have shown severe cases with the presence of SARS-CoV-2 RNA in esophageal ulcers as well as in stomach, duodenum, and rectal tissues of these patients^10^. It was also observed that patients with severe disease are more likely to have abdominal pain when compared to non-severe patients as well as a greater chance of having abnormal serum levels of biomarkers related to liver function and lesion, associated with GI involvement and worse disease prognosis^11^ ^,^ ^12^. Studies have suggested that the angiotensin-converting enzyme II (ACE2) receptor, which mediates SARS-CoV-2 infection, is expressed in lung AT2 cells as well as in the esophagus upper and stratified epithelial cells and absorptive enterocytes from the ileum and colon. These findings may be associated with GI manifestations^11^ ^,^ ^13^. Moreover, SARS-CoV-2 RNA has been identified in stool specimens and anal or rectal swabs of COVID-19 patients^14^. Notably, some data indicate that the viral RNA may remain detectable in the stool even after negative results from respiratory samples^15^ ^,^ ^16^. Therefore, fecal-oral transmission may be another possible SARS-CoV-2 transmission route, and should be considered in infection control measures^17^. In this systematic review, we analyzed the current international evidence regarding the association between the GI tract and COVID-19.

## METHODS

The criteria recommended by the preferred reporting items for systematic reviews and meta-analyses (PRISMA) checklist were followed to conduct this systematic review^18^.

### Eligibility Criteria

Types of participants: Adults and children diagnosed with SARS-CoV-2 infection confirmed by real-time reverse transcriptase-polymerase chain reaction (RT-PCR), who had concomitant GI symptoms. 

Types of study: Prospective and retrospective studies published in peer-reviewed journals up to July, 2020 that reported epidemiological and clinical data of patients with COVID-19, the prevalence of GI symptoms, and the serum levels of biomarkers related to liver function and injury in these patients were included. The following studies were excluded: studies that did not report GI symptoms, duplicated studies, studies that included patients infected with other coronavirus types, case reports, reviews, meta-analyses, systematic reviews, editorials, small case series (< 15 cases), and clinical trials evaluating medications. Studies that did not have a complete version published as a free full text were excluded. Studies published in English, Spanish, and Portuguese were included.

Types of outcome measures: We collected data evaluating the occurrence of GI symptoms caused by COVID-19 and the serum levels of biomarkers related to liver function and lesion.

Information sources: We surveyed the relevant articles published in English, Spanish, and Portuguese up to July, 2020 in the United States National Library of Medicine (PubMed), Medical Literature Analysis and Retrieval System Online (MEDLINE), Scientific Electronic Library Online (SciELO), Latin American Literature in Health Sciences (LILACS), and Virtual Health Library (BVS) databases. The search terms used for all databases were: (Coronavirus [OR] severe acute respiratory syndrome coronavirus 2 [OR] SARS-CoV-2 [OR] COVID-19 [and] gastrointestinal symptoms [OR] clinical features [OR] clinical manifestations). Due to a large number of publications on the topic and their urgency and importance, we also surveyed potentially important articles published in the New England Journal of Medicine (NEJM), the Journal of the American Medical Association (JAMA), the British Medical Journal (BMJ), Gastroenterology, Gut, and the American Journal of Gastroenterology (AJG) in order to increase the sensitivity of the research. 

Study Selection: The eligibility of the articles was evaluated by three independent reviewers (Da Silva, FAF; Santos, MLC; and Marques, HS). Duplicated articles were excluded. The titles and abstracts of the articles were evaluated, and studies that did not fit the inclusion criteria were excluded. A fourth reviewer (de Melo, FF) resolved any disagreements between the three reviewers. In order to verify if the articles met all previously established criteria, each article was individually analyzed. 

Data Collection Process: We developed a structured data extraction spreadsheet specifically for this review based on the criteria recommended by the Cochrane Handbook of Systematic Reviews for Interventions^19^. We independently reviewed the relevant study data and results of interest such as GI symptoms and biomarkers related to liver function and lesion in COVID-19 patients. 

Data items: Information was extracted from each study and stratified into (1) general epidemiologic and clinical characteristics of participants and studies; (2) diarrhea; (3) nausea; (4) vomiting; (5) abdominal pain; (6) any GI symptom; (7) severity of COVID-19 infection; and (8) biomarkers related to liver function and lesion: albumin, prothrombin time, aspartate aminotransferase, and alanine aminotransferase.

Assessment of quality of studies: To assess of the quality of the 43 selected studies, National Institute of Health (NIH/NHLBI) tools, which were developed through a collaboration with the National Heart, Lung, and Blood Institute (NHLBI) and the Research Triangle Institute International, were used^20^. To comply with the aim of this systematic review, the NIH tool for case series was applied in 33 studies. It uses nine domains, including the presence of a clearly defined objective and well-described results. Based on that, each case series received a general classification as long as it received a “yes” in each domain. Good, regular, and bad studies had positive results in ≥ 6 domains, 3-5 domains, and < 3, respectively. For nine studies, the NIH tool for observational cohort and cross-sectional studies was used, which features fourteen domains. Therefore, good, regular, and bad studies obtained “yes” in ≥ 9 domains, 5-8 domains, and ≤ 4 domains, respectively. One study was assessed using the NIH tool for case-control studies, which uses twelve domains, and good, regular, and bad studies obtained “yes” in ≥ 8 domains, 5-7 domains, and ≤ 4 domains, respectively.

## RESULTS

### Study Selection

**FIGURE 1:**
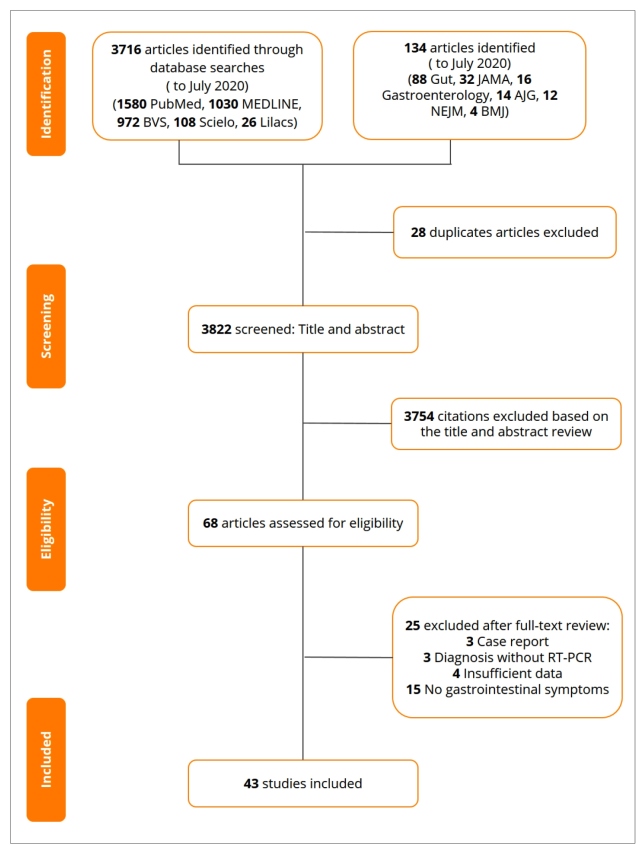
Summary of study selection process.

A total of 3,850 articles were identified in our searches. We excluded 28 duplicate articles, and 3,821 remained. A further 3,754 studies were removed after reviewing the titles and abstracts. The remaining 68 articles were assessed for eligibility, of which 25 were excluded because of the following reasons: three were case reports; three studies reported COVID-19 cases without RT-PCR confirmation; four articles had insufficient data; and 15 studies had no patients experiencing GI symptoms. Finally, 43 studies were included. Figure 1 shows the selection and distribution of articles according to the databases searched from the first search to the application of all the selection criteria.

### Study characteristics

**FIGURE 2:**
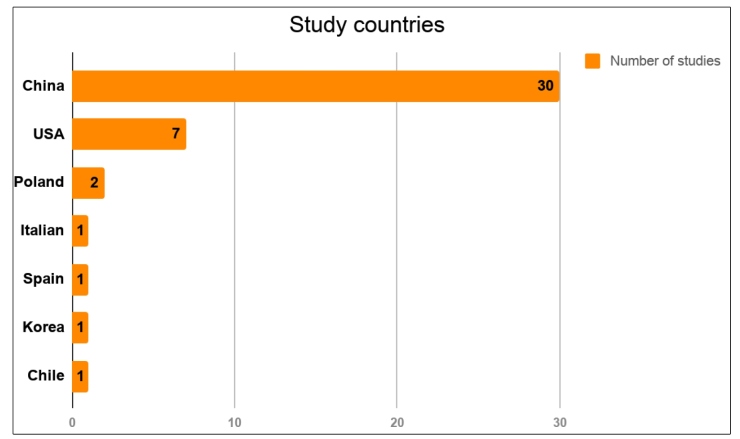
Geographical distribution of the studies.

The characteristics of the 43 studies selected are summarized in Table 1. A total of 18,246 patients, of all age ranges, were included. Most studies were retrospective. Regarding the geographic distribution of the studies, 69% of the articles were from China, 16% were from the USA, 7% were from Poland, and 2% were from Italy, Chile, Spain, and Korea. Figure 2 shows the geographic distribution of the studies. In addition, the articles included had several aims, such as evaluating epidemiological characteristics, imaging, and clinical features, in addition to assessing the occurrence of GI symptoms in patients infected with SARS-CoV-2.

### GI manifestations

**FIGURE 3:**
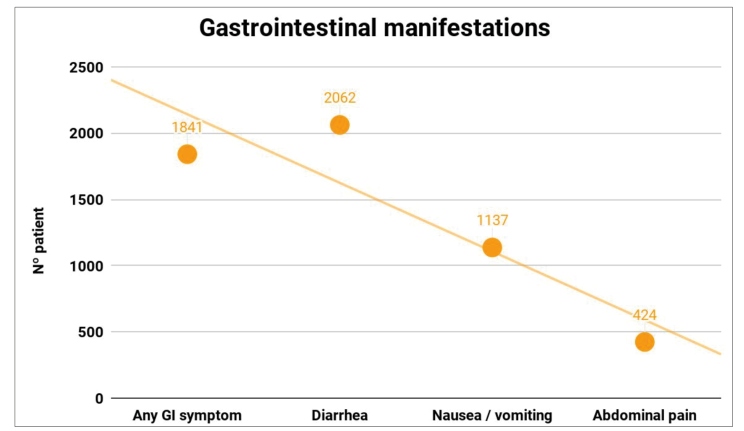
Scatter plot of gastrointestinal symptoms in patients with COVID-19. **Legend:** Graph showing the number of COVID-19 positive patients and the gastrointestinal symptoms seen in 18,246 analyzed patients.

All 43 articles analyzed reported at least one GI symptom in COVID-19 patients. There was no relevant difference in the number of patients between sexes (50.5% of the individuals were men). That percentage did not undergo a substantial change (52.1%) when articles that exclusively reported COVID-19 patients with GI symptoms were included, and a total of 4,614 patients in eight studies were separately analysed^31^ ^-^ ^35^ ^,^ ^51^ ^,^ ^52^ ^,^ ^54^. Diarrhea was the most commonly reported symptom, being detectable in 11.5% (n = 2115) of patients (38 articles), followed by nausea and vomiting, reported in 6.3% (n = 1158) of participants (31 studies), and abdominal pain, found in 2.3% (n = 424) of the individuals (21 studies). In 21 studies, the presence of any GI manifestations showed a prevalence of 30.5% (n = 1841) (Figure 3). In addition to the symptoms shown in Table 1, loss of appetite, anosmia, ageusia, and intestinal bleeding were reported.

**TABLE 1:** Epidemiological and clinical data of positive COVID-19 patients.

N	Author	Country / Year	Study design	N	Adult / Children /	Woman /	Severe / Non	Patients with any	Diarrhea	Nausea / vomiting	Abdominal
					Median Age	Man (N)	Severe (N)	GI symptom (N)	(N)	(N)	pain
1	Chen, *et al.* ^21^	China - 2020	RS	21	Adult: 61 years	4/17	11/10	NR	4	NR	NR
2	Xu, *et al.* ^22^	China - 2020	RS	90	Adult: 50 years	51/39	NR	NR	5	7	NR
3	Li, *et al.* ^23^	China - 2020	RS	83	Adult: 45.5 years	39/44	58/25	7	NR	NR	NR
4	Yang W, *et al.* ^24^	China - 2020	RS	149	Adult: 45.11 years	68/81	NR	NR	11	2	NR
5	Liu, *et al.* ^25^	China - 2020	RS	137	Adult: 57 years	76/61	NR	NR	11	NR	NR
6	Wu, *et al.* ^26^	China - 2020	RS	80	Adult: 44 years	38/42	NR	7	NR	NR	NR
7	Liang, *et al*.^27^	China - 2020	RS	1590	Adult: 48.9 years	674/904	131/-	NR	57	80	NR
8	Zheng, *et al*.^28^	China - 2020	RS	25	Children: 3 years	11/14	2/23	NR	3	2	2
9	Lokken, *et al.* ^29^	USA - 2020	RS	46	Adult: 29 years	46/0	NR	NR	3	5	NR
10	Wang, *et al.* ^30^	China - 2020	RS	275	Children/Adult: 49 years	147/128	45/230	NR	7	8	NR
11	Jin, *et al.* ^31^	China - 2020	PS	651	Adult: 46.14 years	320/331	64/-	74	53	21	NR
12	Lin, *et al.* ^32^	China - 2020	PS	95	Adult: 45.3 years	50/45	20/75	23	23	21	NR
13	Redd., *et al.* ^33^	USA - 2020	PS	318	Adult: 63.4 years	144/174	NR	195	107	133	46
14	Sierpiński, *et al.* ^34^	Poland - 2020	RS	1942	Adult: 50 years	1169/773	NR	912	470	NR	NR
15	Luo, *et al.* ^35^	China - 2020	RS	1141	Adult: 53.8 years	NR	NR	263	68	253	45
16	Liu BM, *et al.* ^36^	China - 2020	RS	68	Adult: 44.3 years	43/25	NR	NR	5	4	NR
17	Li, *et al.* ^37^	China - 2020	RS	70	Adult: 44.6 years	23/43	NR	2	2	2	0
18	Yin, *et al.* ^38^	China - 2020	RS	33	Adult: 46 years	17/16	NR	NR	5	NR	NR
19	Derespina, *et al.* ^39^	USA- 2020	RS	70	Children: 15 years	27/42	NR	NR	18	24	NR
20	Xiong, *et al.* ^40^	China - 2020	PS	244	Children: 1.2 years	94/150	11/-	8	15	23	4
21	Pan, *et al.* ^41^	China - 2020	RS	204	Adult: 52.9 years	97/107	NR	81	35	4	2
22	Du, *et al.* ^42^	China - 2020	RS	182	Children: 6 years	62/120	4/-	20	9	7	7
23	Rivera, *et al.* ^43^	Spain - 2020	RS	76	Adult: 45.8 years	53/23	NR	57	31	24	21
24	Zhang, *et al.* ^44^	China - 2020	RS	140	Adult: 57 years	69/71	11/31	8	NR	NR	NR
25	Kim, *et al.* ^45^	Korea -2020	RS	28	Adult: 40 years	13/15	NR	3	3	1	1
26	Zhao, *et al.* ^46^	China - 2020	RS	101	Adult: 44 years	45/56	14/-	5	3	2	NR
27	Xu, *et al.* ^47^	China - 2020	RS	62	Adult: 41 years	27/35	NR	5	3	NR	NR
28	Yang, *et al.* ^48^	USA- 2020	RS	124	Adult: 75.7 years	66/58	NR	NR	9	14	NR
29	Suleyman, *et al.* ^49^	USA- 2020	RS	463	Adult: 57.5 years	259/204	NR	NR	100	147	NR
30	Chen, *et al.* ^50^	China - 2020	RS	175	Adult: 45 years	87/88	40/-	NR	35	7	5
31	Cholankeril, *et al.* ^51^	USA -2020	RS	116	Adults: 50 years	54/62	NR	59	12	12	10
32	Nobel YR, *et al.* ^52^	USA -2020	RS	278	NR	133/145	NR	97	56	63	NR
33	Wei, *et al.* ^53^	China - 2020	RS	84	Adults: 37 years	56/28	NR	4	26	22	2
34	Xiao F, *et al*.^54^	China - 2020	PS	73	Adults: 43 years	32/41	NR	10	26	NR	NR
35	Díaz LA, *et al.* ^55^	Chile - 2020	PS	7016	Adults: 39.7 years	3508/3508	NR	NR	511	NR	260
36	Chen T, *et al.* ^56^	China - 2020	RS	274	Adults: 62 years	103/171	274/-	NR	77	40	19
37	Argenziano *et al.* ^57^	USA - 2020	RS	1000	Adults: 63 years	404/596	NR	NR	236	178	NR
38	Zheng, *et al.* ^58^	China - 2020	RS	52	Children: 9 years	24/28	NR	1	NR	NR	NR
39	Garazzino, *et al*.^59^	Italian - 2020	RS	168	Children: 5.2 years	74/94	NR	NR	22	9	NR
40	Wang, *et al.* ^60^	China - 2020	RS	125	Adult: 38.76 years	54/71	NR	NR	50	24	NR
41	Du, *et al.* ^61^	China - 2020	RS	67	Children/Adult: 34.10 years	35/32	NR	NR	2	4	0
42	Nowak, *et al.* ^62^	Poland- 2020	RS	169	Adult: 63.7 years	82/87	NR	NR	8	6	NR
43	Chen, *et al.* ^63^	China - 2020	RS	141	Adult: 47.3 years	68/73	15/-	NR	5	9	NR

**RS:** Retrospective study; **PS:** Prospective study; **NR:** not reported; **GI:** gastrointestinal.

### Subgroup analyses

In order to examine the possible relationship between the presence of GI symptoms and COVID-19 severity, we analyzed the illness seriousness of the patients present in the 43 articles included. Among them, 14 studies, shown in Table 1, stratified patients as severe/critical or not severely ill. A total of 17.5% (700) of the patients were considered to have severe COVID-19, whereas 9.8% (394) had a non-severe illness. We also observed that the average age among severely ill adults ranged from 44 to 62 years, whereas the mean age among children who experienced severe disease ranged from 1.2 to 6 years.

### Liver function and injury

Among the 43 studies included in the final analysis, 24 evaluated biomarkers related to liver function and injury; however, we only analyzed articles that assessed those biomarkers in COVID-19 patients with GI symptoms. In this regard, we included data from seven articles with a total of 665 patients (Table 2), from which two studies with 209 patients reported mild increases in the mean aspartate aminotransferase (AST) and alanine aminotransferase (ALT) serum levels^35^ ^,^ ^51^

**TABLE 2:** Data on the serum level of biomarkers related to liver function and injury in patients with COVID-19.

Author	N	AST (IU/L)		ALT (IU/L)		Albumin (g/L)		Prothrombin time (s)	
		Value	P	Value	P	Value	P	Value	P
Jin, *et al.* ^31^	74	29.35	0.02	25.0	0.203	40.13	0.039	NR	NR
Lin, *et al.* ^32^	58	17.6 ± 5.6	NR	22.5 ± 19.2	NR	NR	NR	NR	NR
Redd, *et al.* ^33^	195	46.7 ± 35.3	0.26	35.9 ± 31.8	0.97	NR	NR	35.8 ± 11.6	0.52
Luo, *et al.* ^35^	183	65.8 ± 12.7	NR	66.4 ± 13.2	NR	NR	NR	NR	NR
Pan, *et al.* ^41^	103	35.12 ± 6.58	0.032	42.24 ± 43.83	0.011	36.16 ± 6.49	0.707	13.13 ± 1.88	0.024
Cholankeril, *et al.* ^51^	26	64	0.009	59	0.009	NR	NR	NR	NR
Wei, *et al.* ^53^	26	24.9 ± 6.4	0.055	20.6 ± 7.5	0.014	40.5 ± 4.7	0..837	13.8 ± 2.6	0.051

AST range = 15-40 IU/L; ALT range = 9-50 IU/L; albumin range= 40-55 g/L; prothrombin time range= 11-13.5 s.

### Assessment of quality of studies

**FIGURE 4:**
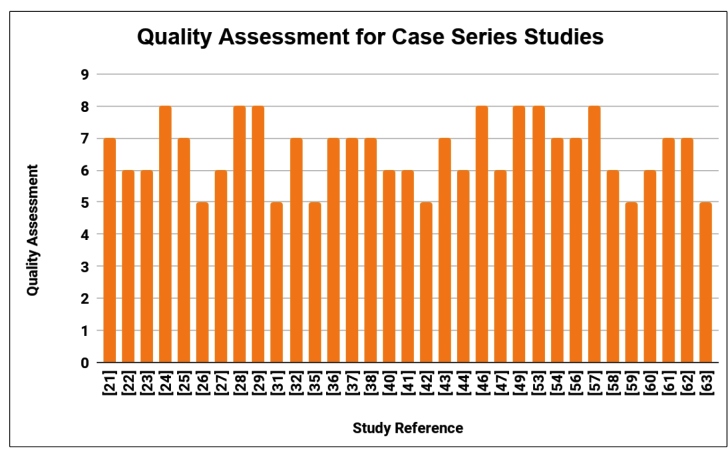
Quality analysis chart of included studies.

The quality of the studies was assessed using NIH tools for case series^20^ in 33 studies, and the results are shown in Figure 4. The scores were: 8/9 for 7 studies (22%), 7/9 for 11 studies (33%), 6/9 for 9 studies (27%), and 5/9 for 6 studies (18%). Thus, 27 studies (82%) were of good quality (score ≥ 6), 6 studies (18%) of regular quality (score 3-5), and no study was found to be of poor quality. Nine studies^30^ ^,^ ^33^ ^,^ ^34^ ^,^ ^39^ ^,^ ^45^ ^,^ ^48^ ^,^ ^50^ ^,^ ^51^ ^,^ ^55^ included in this systematic review were analyzed using NIH tools for observational cohort and cross-sectional studies^20^. The scores were as follows: 10/14 for two studies (22.2%), 9/14 for two studies (22.2%), 8/14 for one study (11.2 %), 7/14 for three studies (33.2%), and 6/14 for one study (11.2%). Thus, four studies (44,4 %) had a good quality (score ≥ 9) and 5 studies (55.6 %) had regular quality (score 5-8). The case-control study^52^ included was analyzed according to NIH tools for case-control studies^20^ and obtained a 7/12 score, which was considered as a regular-quality study. 

## DISCUSSION

Since the first infection cases reported in December 2019, SARS-CoV-2 has spread worldwide and, subsequently, COVID-19 was declared a pandemic by the World Health Organization^64^ ^,^ ^65^. Therefore, a large number of studies have been published by the scientific community in a short period of time in order to understand the mechanisms of this new virus and to research possible treatments and vaccines.

The most commonly reported symptoms in clinical and epidemiological studies involving COVID-19 patients are fever, dry cough, and dyspnea^25^ ^-^ ^27^. However, a growing number of studies have reported a series of GI symptoms in these patients due to the involvement of the GI system in the pathophysiology of the COVID-19. 

### Diarrhea

All of the articles included in this systematic review reported patients with diarrhea. Among the studies, 39 provided the number of patients who had that symptom, as shown in Table 1, whereas four articles did not provide its prevalence^23^ ^,^ ^26^ ^,^ ^44^ ^,^ ^58^. Our results demonstrate that diarrhea is the most common GI symptom in SARS-CoV-2 infection, in agreement with a prior meta-analysis that evaluated 26 studies and 4,676 patients^66^. Among the individuals sampled in the present review, 2,115 (11.5%) manifested diarrhea during SARS-CoV-2 infection. A similar prevalence (10.3%) was reported by Cholankeril *et al.* (2020)^51^ in an American study that evaluated 116 patients. In this systematic review, the prevalence of diarrhea ranged from 2.8%^37^ to 40.7%^43^ among studies assessing general epidemiological and clinical characteristics of COVID-19 patients. On the other hand, the percentage of individuals who experienced symptoms varied from 5.95%^35^ to 35.6%^54^ in studies that only included patients with GI symptoms during SARS-CoV-2 infection. With regard to the diarrhea duration, Jin *et al.* reported an average period of 4 days in 53 patients, ranging from 1 to 9 days, with a self-limited course. Some authors have studied the relationship between GI symptoms and ACE2 expressed on AT2 cells of the GI system, which may allow SARS-CoV-2 infection^11^ ^,^ ^13^ ^,^ ^67^. Adding to this knowledge, in a meta-analysis that included 4,243 patients with COVID-19 and GI symptoms, SARS-CoV-2 RNA was detected in stool samples of 48.1% (95% confidence interval [CI]: 6.9-36.7) of the participants^68^. Moreover, it should be emphasized that some authors reported that the first COVID-19 symptom can be a GI presentation, as observed in eight patients from a relevant study who had fever and diarrhea before the onset of respiratory manifestations^69^. Therefore, health professionals should not rule out a COVID-19 diagnosis in patients with diarrhea in geographical areas with SARS-CoV-2 circulation. 

### Nausea and vomiting

Our analyses showed that 1,158 (6.3%) of the patients presented with nausea and/or vomiting in 31 studies, as described in Table 1. These data are similar to the results of Chen *et al.* (2020)^21^ and Liang *et al.* (2020)^27^ who demonstrated a prevalence of nausea and/or vomiting of 6.3% (9/141) and 5% (80/1590), respectively. In a relevant review that included 2,023 patients, it was observed that the presence of vomiting was more common in children than in adults, with 6.5%-66.7% and 3.6%-15.9% prevalence ranges, respectively^9^. This phenomenon was verified by our review, since Lokken *et al*. (2020)^29^ and Argenziano *et al.* (2020)^57^ reported nausea and/or vomiting prevalence rates of 10.8% and 17.8% in an adult population, whereas Redd *et al.* (2020)^33^ and Derespina *et al.* (2020)^39^ found these symptoms in 41.8% and 34.2% of SARS-CoV-2-infected children, respectively.

### Abdominal pain

The prevalence of abdominal pain in our analysis was 2.3% (424) in 21 studies. In a meta-analysis comprising 4,243 patients, it was observed that 17.1% of the patients with severe COVID-19 had GI symptoms (95% CI = 6.9-36.7)^68^ . Interestingly, another meta-analysis observed that critically ill COVID-19 patients have significantly higher odds of experiencing abdominal pain when compared to not severely ill patients (OR = 7.17, 95% CI = 1.95-26.34, P = 0.003), and that symptoms may be a predictor of unfavorable outcomes^11^ . In order to increase the level of evidence on the relationship between abdominal pain and SARS-CoV-2 infection prognosis, further studies should be performed.

### Liver function and damage

In our results, seven authors evaluated biomarkers related to liver function and damage in patients with COVID-19 and GI symptoms (Table 2). However, only the studies by Luo *et al*. (2019)^35^ and Cholankeril *et al.* (2020)^51^ reported abnormal AST and ALT averages. The latter observed an association between the severity of the disease and AST levels (Pearson’ s coefficient = 0.33; P = 0.009). In a Chinese meta-analysis of 6,686 patients, a significant increase was observed in both ALT (OR = 1.89, 95% CI = 1.30-2.76, P = 0.0009) and AST (OR = 3.08, 95% CI = 2.14-4.42, P < 0.00001) levels among severely ill patients than in non-severely ill individuals^70^ . In addition, an interesting meta-analysis from Canada with 3,615 adult patients diagnosed with COVID-19 from 15 studies noted that acute liver injury was associated with increased mortality (RR = 4.02 [1.51, 10.68], P = 0.005)^71^ . Liver abnormalities and the subsequent increase in the circulating levels of cytolysis biomarkers in patients with COVID-19 may be caused by the infection-associated inflammatory storm, hepatic ischemia, reperfusion dysfunction, or drug toxicity^72^ . In fact, AST has been considered as a hepatic marker of COVID-19 severity; however, we understand that such enzymes have high activities in the liver, heart, and muscles, in addition to minimal activity in the kidney and pancreas. In view of the association of SARS-CoV-2 with extrapulmonary manifestations such as cardiac repercussions^73^ , for example, the increase in AST rates may not be such a sensitive marker for liver injury in this context. ALT also plays a role in various organic systems; nonetheless, it has the greatest activity in the liver^74^ . A remarkable study on liver enzymes concluded that restricting the biological role of these enzymes to liver damage is an underestimated interpretation of these biomarkers^75^ . In addition to liver injury biomarkers, work should begin to further analyze liver function biomarkers to build a global view of the consequences of SARS-CoV-2 infection at the liver level.

### Study limitations

We aimed to limit publication bias by including studies published in languages other than English. However, this systematic review has some limitations. First, this study was mostly a compound of retrospective studies. Moreover, there is a potential risk of heterogeneity and publication bias with regard to COVID-19 patients with GI symptoms, as well as to the disease severity criteria used by the authors. 

In conclusion, our results suggest that digestive symptoms are common in COVID-19 patients. In addition, alterations in cytolysis biomarkers could also be observed in a lesser proportion, calling attention to the possibility of hepatic involvement in SARS-CoV-2-infected individuals.

## ARTICLE HIGHLIGHTS

### Research background

The pandemic caused by severe acute respiratory syndrome coronavirus 2 (SARS-CoV-2) infection has greatly challenged public health worldwide. COVID-19 is currently described as a disease with a broad spectrum of symptoms, the most prevalent being dry cough, fever, and shortness of breath. However, a growing number of studies have reported GI symptoms such as diarrhea, nausea, vomiting, abdominal pain, anorexia, and GI bleeding, calling attention to the importance of this set of clinical manifestations among infected individuals.

### Research motivation

SARS-CoV-2 has spread worldwide, and as at the last week of July 2020, more than 16 million cases and 662 thousand deaths have been reported globally. In this scenario, it is important to identify the diversity of clinical manifestations of COVID-19, understanding the different ways through which patients can be affected. In this sense, understanding the association between COVID-19 and GI symptoms is crucial.

### Research objectives

To perform a systematic review of the GI symptoms and serum levels of cytolysis biomarkers related to liver function and injury among COVID-19 patients.

### Research methods

A systematic review of the current literature as at July, 2020 was performed according to the PRISMA statement. During the screening process, articles that were not published in English, Portuguese, or Spanish as well as unavailable reports and single case reports were excluded. The search was performed using a combination of the terms Coronavirus [OR] severe acute respiratory syndrome coronavirus 2 [OR] SARS-CoV-2 [OR] COVID-19 [and] gastrointestinal symptoms [OR] clinical features [OR] clinical manifestations. The databases selected for this review were PubMed, MEDLINE, SciELO, LILACS, and BVS. Potentially important articles published in NEJM, JAMA, BMJ, Gastroenterology, Gut, and AJG were also selected. 

### Research results

This systematic review included 43 studies, including 18,246 patients. There was no significant difference between the number of male (50.5%) and female (49.5%) participants. Individuals of all age groups were included. At least one patient in each study included had GI symptoms associated with COVID-19, and the prevalence of such symptoms was similar among men and women (52.1% and 49.5%, respectively). Diarrhea was the most common GI symptom, affecting 11.5% of the patients, followed by nausea and vomiting (6.3%) and abdominal pain (2.3%). Loss of appetite, anosmia, ageusia, and GI bleeding were also reported. With regard to clinical severity, 17.5% of the patients were classified as severely ill, whereas 9.8% of them were considered to have a non-severe disease. Moreover, the mean age of severely ill patients ranged from 44 to 62 years in adults and from 1.2 to six years among children. Some studies evaluated cytolysis biomarkers in COVID-19 patients who had GI symptoms, showing increased aspartate transaminase and alanine aminotransferase levels in a portion of the 209 analyzed patients and two studies.

### Research conclusions

This systematic review shows that COVID-19 patients often experience GI symptoms and suggests a potential relationship between the presence of these symptoms and increased disease severity. Moreover, alterations in cytolysis biomarkers could also be observed in a lesser proportion, calling attention to the possibility of hepatic involvement in SARS-CoV-2-infected individuals.

### Research perspectives

The information gathered by this systematic review provides an update on COVID-19 GI manifestations and may be useful for clinical practitioners in the management of COVID-19 patients. Moreover, it adds to the understanding of the disease and should be considered in further studies evaluating the repercussions of SARS-CoV-2 infection on the human digestive system.
